# Free Fatty Acid-Induced Peptide YY Expression Is Dependent on TG Synthesis Rate and Xbp1 Splicing

**DOI:** 10.3390/ijms21093368

**Published:** 2020-05-10

**Authors:** Chad M. Paton, Yura Son, Roger A. Vaughan, Jamie A. Cooper

**Affiliations:** 1 Department of Foods & Nutrition, University of Georgia, Athens, GA 30602, USA; yura.son@uga.edu (Y.S.); jamie.cooper@uga.edu (J.A.C.); 2 Department of Food Science & Technology, University of Georgia, Athens, GA 30602, USA; 3 Department of Nutritional Sciences, Texas Tech University, Lubbock, TX 79409, USA; rvaughan@highpoint.edu

**Keywords:** satiety, lipogenesis, PYY, lipid metabolism, obesity

## Abstract

Gut-derived satiety hormones provide negative feedback to suppress food intake and maintain metabolic function in peripheral tissues. Despite the wealth of knowledge of the systemic effects of these hormones, very little is known concerning the mechanisms by which nutrients, such as dietary fats, can promote the expression of genes involved in L-cell hormone production. We have tested the role of various dietary fats and found that after hydrolysis into free fatty acids (FFA’s), there is a differential response in the extent to which they induce PYY gene and protein production. The effect of FFA’s also seems to relate to triglyceride (TG) re-esterification rate, with MUFA re-esterifying faster with lower PYY production. We have also found that there are differences in potency of FFA’s based on their desaturation patterns in vitro. The potency effect of FFA’s is influenced by the rate of TG re-esterification, such that the longer FFA’s are in contact with L-cells, the more PYY they produce. We found that chronic consumption of high-fat diets enables the small intestine to re-esterify FFA’s into TG faster and earlier which resulted in a blunted postprandial PYY response. Lastly, we found that FFA’s induce X-box-binding protein-1 activation (Xbp1s) in L-cells and that adenoviral delivery of Xbp1s was sufficient to induce PYY gene expression. Taken together, the present work indicates that dietary fat can induce satiety, in part, prior to re-esterification. Chronic high-fat diet consumption increases the rate of re-esterification which diminishes satiety and may lead to increased food intake. Targeting intestinal TG synthesis may prove beneficial in restoring obesity-associated reductions in postprandial satiety.

## 1. Introduction

Peptide tyrosine tyrosine (PYY) is a peptide hormone produced and secreted by enteroendocrine L-cells of the ileum and colon in response to nutrient supply. When released from L-cells, PYY reduces food intake through inhibitory signaling via neuronal receptors in the arcuate nucleus of the hypothalamus. In the pancreas, PYY improves insulin release and reduces exocrine pancreatic secretions. In the gut, PYY slows gastric motility, slows absorption of macro- and micronutrients, and produces systemic feelings of fullness [1,2]. A major nutrient that induces PYY release and satiety is dietary fat, with varying degrees of PYY release based on fatty acid composition [3,4]. Despite the knowledge of PYY’s effects on tissues beyond the L-cell, nothing is known regarding how nutrient–gene interactions produce PYY at the mRNA level.

What is known to-date regarding the mechanism of PYY production (e.g., expression or secretion) is essentially limited to FFA-mediated cell signaling [5] or extrapolated from other nutrient–hormone results [6,7,8,9]. There are only a handful of studies that have looked at the role of intestinal triglyceride (TG) metabolism and its relationship with satiety hormone production. In particular, the role of Acyl CoA:diacylglycerol acyltransferase-1 (DGAT1) inhibition on PYY or glucagon-like peptide (GLP)-1, satiety, and food intake was investigated previously [10,11,12,13,14]. DGAT1 knockout mice displayed delayed gastric emptying following triglyceride (TG) gavage and GLP-1 and PYY were both elevated 2-h post gavage compared to wild-type mice [14]. Additionally, targeted inhibition of DGAT1 (DGAT1i) with small molecule inhibitors has consistently shown that gastric emptying rate is decreased, plasma TG appearance rate is decreased, and PYY and GLP-1 are both increased acutely [11]. In chronic DGAT1i studies, food intake and body weights are lower, which has been attributed at least in part, to improved satiety but the mechanisms governing these approaches is not known [11,12,13].

Nutrient–gene interaction is a critical aspect of maintaining physiological homeostasis. It enables the organism to adapt to acute and chronic changes in metabolic flux by altering transcriptional pathways responsible for assimilating nutrients. Dietary fat has been shown to induce endoplasmic reticulum (ER) stress, with varying degrees of activation based on free fatty acid (FFA) composition [15,16,17]. Essentially, when cells are overloaded with nutrients, including carbohydrates and fat, the ER senses the influx and through a p38/c-Jun N-terminal kinase (JNK) pathway, inositol requiring enzyme-1α (Ire1α)-mediated splicing of X-box-binding protein-1 (Xbp1) occurs. Xbp1 mRNA is present in the cytosol in an unspliced form (Xbp1u) that contains a 23 nucleotide (nt) intron with an early termination (stop) codon. When Ire1α-mediated splicing occurs, the 23nt intron is spliced out which removes the stop codon and generates Xbp1-spliced (Xbp1s). When translated, the Xbp1s protein is a transcription factor with targets that include genes involved in lipogenesis, glucose metabolism, adipogenesis, and ER stress resolution targets.

The purpose of the current study was to begin to define the nutrigenetic mechanisms by which FFA’s induce PYY gene and protein expression to better understand the physiology and biochemistry of diet-gene interactions in L-cells. We found that that the longer FFA’s are in contact with intestinal L-cells, the more PYY is produced. Additionally, we hypothesized that PYY gene expression is controlled by FFA-induced Xbp1 splicing in L-cells. Direct delivery of Xbp1s into L-cells increased PYY gene and protein expression and blocking the cell stress response prevented FFA-induced PYY gene expression.

## 2. Results

**Figure 1:**
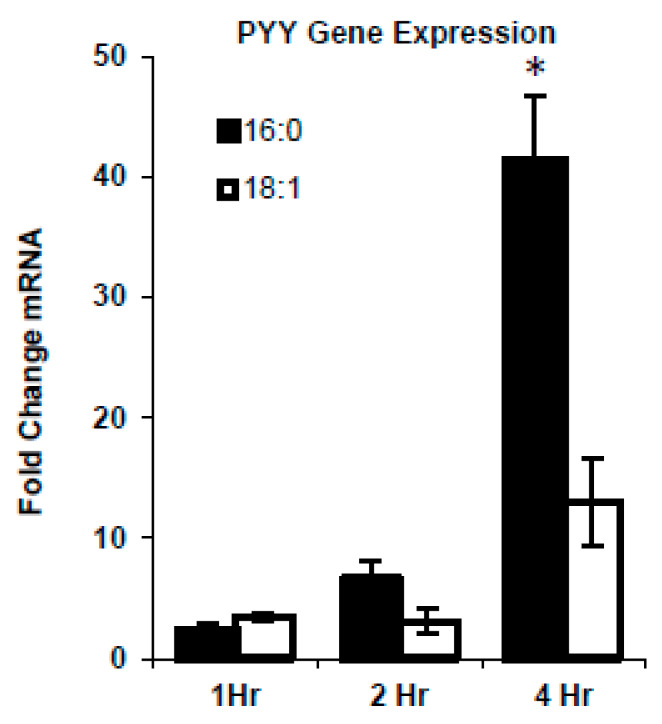
MUFA vs. SFA induced PYY response in vitro. STC-1 cells were treated with vehicle (EtOH), MUFA (18:1), or SFA (16:0), and PYY mRNA levels were measured by qPCR. Both SFA and MUFA increased PYY level in L-cells in a time-dependent manner. However, SFA treatment induced a higher level of PYY than MUFA. Data are represented as mean ± SEM; * *p* < 0.05 vs. 18:1.

We knew from our previous work that fatty acid composition differentially affected PYY responses [3,4,18,19]. Therefore, we first attempted to determine the impact of SFA versus MUFA on PYY gene expression in the STC-1 L-cell line. FFA’s were provided to cells from 1–4 h and gene induction was determined. As expected, the longer the FFA’s were in contact with the STC-1 cells, the greater the increase in PYY expression (Figure 1). There were no differences between SFA and MUFA from 0–2 h. However, after 4-h, there was a greater degree of induction with SFA treatment versus MUFA (41.4 ± 5.4 vs. 19.4 ± 8.0 fold; *p* = 0.01).

**Figure 2:**
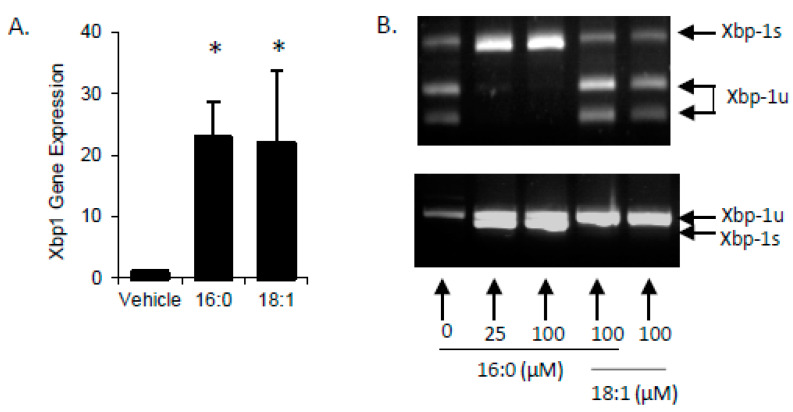
SFA but not MUFA increases Xbp1 splicing. (**A**) Accumulation of FFA have been shown to activate the unfolded protein response with specific induction of Xbp1s. Both SFA (16:0) and MUFA (18:1) increased Xbp1 gene expression after treatment for 2-h. (**B**) Xbp1 exists as either inactive, unspliced (Xbp1u), active, spliced (Xbp1s), or a combination of the two. Xbp1 staus can be examined by resistance to (Xbp1u) or digestion with *Pst*I (Xbp1s) or by size (Xbp1u is 26 nucleotides longer than Xbp1s). Cells treated with 25- or 100 µM 16:0 displayed all Xbp1 mRNA as Xbp1s and 18:1 treated cells, either with or without 16:0 showed a marked reduction in Xbp1s content. Data are represented as mean ± SEM; * *p* < 0.05 vs. vehicle.

Accumulation of FFA’s in cells has been shown to activate the unfolded protein response (UPR) with specific induction of X-box-binding protein-1 (Xbp1) activation via splicing (Xbp1s). In STC-1 cells treated with SFA or MUFA, there was a significant increase in total Xbp1 mRNA (Xbp1 + Xbp1s) (Figure 2A). Upon further examination, we found that unspliced Xbp1 (Xbp1u) increased with MUFA treatment whereas Xbp1s increased with SFA. It is important to note that Xbp1u does not encode for a functional transcription factor due to the presence of the in-frame stop codon; when the stop codon is removed via RNA splicing, the transcriptionally active Xbp1s is functional. At low (25 µM) and high (100 µM) concentrations, SFA increased Xbp1s with no impact on the UPR from high MUFA. When combined with MUFA, high SFA failed to induce Xbp1s (Figure 2B) which is in agreement with previously published work indicating that intracellular FFA esterification rate (FFA→TG) is likely to contribute to the degree of Xbp1s [20], which may lead to UPR activation in L cells.

**Figure 3:**
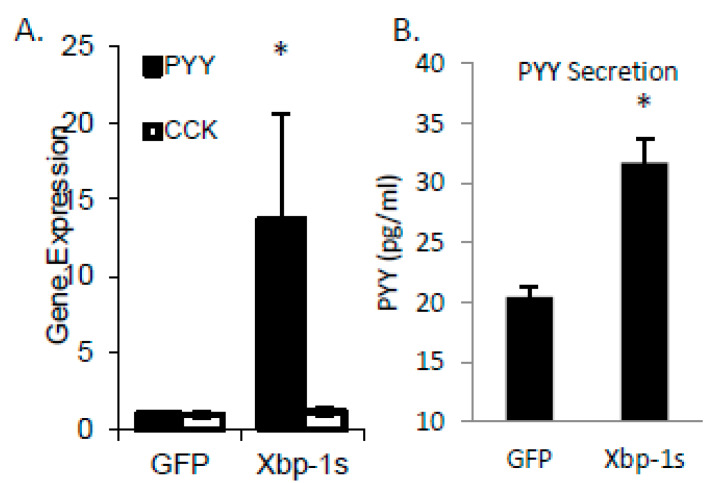
Lipid-mediated regulation of PYY gene expression through Xbp1s. (**A**) Using pre-formed Xbp1s as adenovirus, cells receiving active Xbp1s increased PYY gene expression with no change in CCK gene induction. (**B**) With Xbp1s adenovirus treatment of STC-1 cells, PYY secretion into the media increased from 20.5 ± 0.8 (with GFP control adenovirus) to 31.7 ± 2.1 *p* = 0.007. Data are represented as mean ± SEM; * *p* < 0.05.

**Figure 4:**
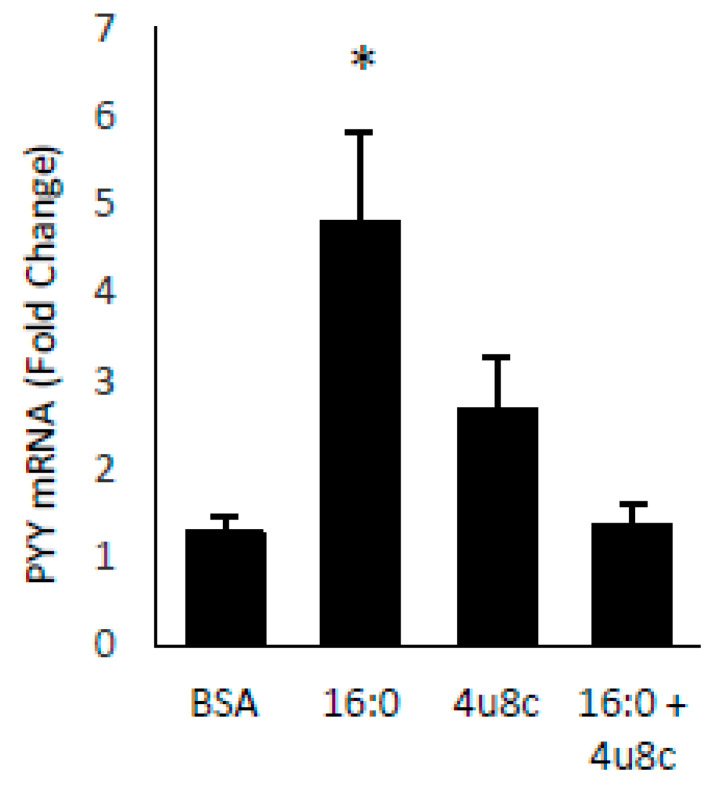
Blocking Xbp1 splicing prevents FFA-induced PYY expression. STC-1 cells were treated with SFA (16:0), the Ire1α inhibitor 4μ8c, or both, and PYY gene expression was measured. SFA induced PYY in the presence of Ire1α activity (which induces Xbp1s) but not in the absence of activity (which prevents Xbp1s). Data are represented as mean ± SEM; * *p* < 0.05.

SFA treatment of STC cells induced both PYY expression and Xbp1s. To demonstrate a direct effect of Xbp1s on PYY gene expression and protein secretion, we treated cells with adenovirus expressing active Xbp1s. Gene expression of PYY was increased 13.7 ± 7.0 fold (*p* = 0.05) with no significant effect on the cholesytokinin (CCK) expression demonstrating specificity of Xbp1s for PYY (Figure 3A). Media from the adenovirus treated cells was collected and PYY protein was measured by RIA with a significant increase in PYY levels in Xbp1s treated cells (GFP = 20.4 ± 0.8 pg/mL vs. Xbp1s = 31.7 ± 2.0 pg/mL; *p* = 0.007) (Figure 3B). Next, to confirm the necessity of Xbp1s activation for FFA-mediated PYY expression, STC-1 cells were then treated with SFA, 4µ8c (an Ire1α inhibitor), or SFA + 4µ8c for 1-h and PYY gene expression was determined (Figure 4). Since SFA showed a consistent ability to activate both PYY gene expression and Xbp1s, we wanted to uncouple these two processes in order to determine if SFA-mediated Xbp1s activation was required for SFA-induced PYY expression. Therefore, in the absence of Ire1α activity (and subsequent Xbp1 activation), SFA would not be able to induce PYY gene expression if Xbp1s was necessary. As expected, SFA increased PYY gene expression (4.8 ± 1.0 fold; *p* = 0.004) with no significant effect from 4µ8c alone. However, when Ire1α-mediated Xbp1s was blocked via 4µ8c, SFA failed to induce PYY expression (1.4 ± 0.4 fold; *p* = 0.8).

**Figure 5:**
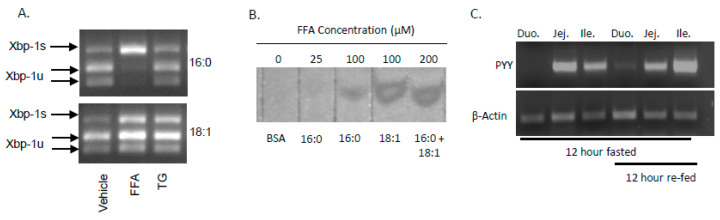
SFA as FFA vs. TG and its effect on Xbp1s and TG synthesis. (**A**) STC-1 cells were treated with MUFA (18:1) or SFA (16:0) as FFA or TG and Xbp1s and Xbp1u mRNA levels were measured by PCR. SFA induced Xbp1s only as FFA and no effect was observed with MUFA or TG (SFA or MUFA) on splicing. (**B**) MUFA enhances the amount of TG synthesis as shown by density of TG bands on TLC plates. (**C**) MUFA is absorbed in the proximal small intestine (early in digestion) compared to distal absorption of SFA, whereas PYY expression is in the distal region. This evidence suggests that (1) non-esterified saturated fatty acids are responsible for inducing Xbp1s in the gut, (2) MUFA’s enhance the rate of TG synthesis (i.e., FFA disappearance, and (3) the earlier FFA’s are absorbed (i.e., proximal SI), the less likely they are to reach the distal SI where PYY is expressed.

In addition to the known effects of SFA vs. MUFA on Xbp1 splicing, it is also clear that FFA→TG esterification is impacted by fatty acid composition. We and others have shown that SFA is more slowly incorporated into TG than MUFA [20,21], and this phenomenon may also impact FFA-induced PYY expression. To test this, we first sought to determine the impact of FFA vs. TG on PYY expression. Thus far, all of our in vitro studies of lipid-mediated induction of PYY have focused on FFA, not TG. Therefore, we determined whether TG was capable of inducing Xbp1s in L-cells by treating with 16:0 FFA and its TG species, tripalmitin, or 18:1 FFA and its TG species, triolein. When cells were treated with 16:0 FFA, there was a nearly complete conversion of Xbp1 into Xbp1s with no effect seen from 18:1 FFA (Figure 5A). Tripalmitin and triolein had no effect on Xbp1s indicating that FFA, not TG, induces the UPR in STC-1 cells. The fact that SFA FFA and not MUFA or TG induces PYY is notable, as absorption rate and re-esterification rate is affected by fatty acid composition.

We and others have shown that SFA is poorly incorporated into TG and there is a preferential desaturation activity to convert SFA into MUFA via stearoyl-CoA desaturase-1 (SCD1) in tissues [20,21]. When 25–100 µM 16:0 was added to cells, very little TG was observed by thin layer chromatography (Figure 5B). However, 18:1 alone or with 16:0 generated a greater degree of TG, thus illustrating the beneficial effect of MUFA on TG synthesis. As a result, TG production is faster with dietary MUFA and is absorbed in the proximal small intestine (SI), whereas SFA is absorbed in the distal SI. We sectioned the SI of male, chow-fed C57Bl6/j mice that were either fasted (low PYY expression) or re-fed (high PYY expression) and assessed PYY production in the duodenum, jejunum, or ileum. As expected, PYY expression was restricted to the distal regions of the SI where a greater portion of SFA would be expected to be present (Figure 5C). In light of these results, it would be expected that SFA would induce more satiety by reaching the distal SI because it was not incorporated into TG to the same degree as MUFA.

**Figure 6:**
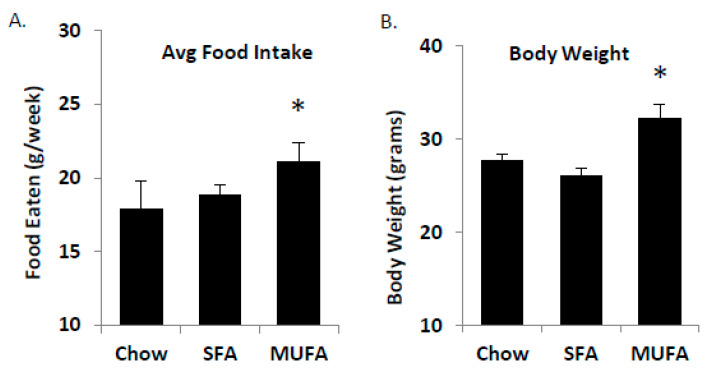
Food intake and body weight changes. Male C57Bl/6 mice (age 8 weeks) were fed ad libitum either Chow, SFA-, or MUFA-enriched diets for 4 weeks. Animals on MUFA-rich diets had the greatest food intake (**A**) and displayed the greatest change in body weight over 4 weeks (**B**). Data are represented as mean ± SEM; *n* = 8 mice/group; * *p* < 0.05.

To test the concept that MUFA’s are absorbed faster than SFA, we fed mice ad libitum diets that were matched in macronutrient content but varying in lipid composition for 4 weeks (Table 1). Food intake was measured weekly and mice receiving the MUFA-enriched diets ate more than the SFA-enriched group (Figure 6A). The food intake was matched by a significant increase in body weight after 4-weeks on the diet (Figure 6B). The food intake and body weight data indicate that a functional difference exists between MUFA and SFA-rich diets, potentially through a differential response in satiety.

**Figure 7:**
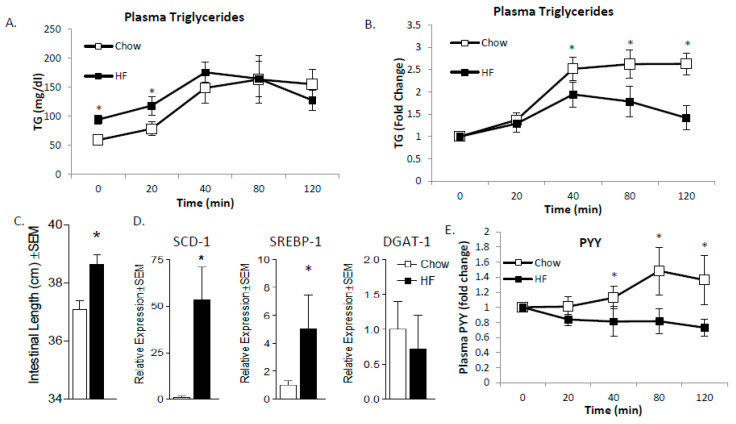
Intestine adaptations to HF diet. Mice were fed chow or high SFA diet for 12-weeks, then gavaged with an SFA-rich meal. Plasma TG following the gavage was measured as absolute (**A**) or relative (**B**). One week later, tissues were collected for analysis where intestinal length was measured and found to be longer in HF-fed mice (**C**). Additionally, lipogenic genes (SCD1 and SREBP1c but not DGAT1) were increased (**D**). High SFA-fed mice had lower satiety response to the gavage as determined by plasma PYY levels (**E**). These data indicate that chronic high SFA diets lead to increased clearance through increased absorptive area and lipogenic capacity which reduces post-meal satiety response. Data are represented as mean ± SEM; * *p* < 0.05.

Finally, we assessed the impact of obesity on MUFA vs. SFA-mediated PYY production in mice. An understudied phenomenon related to HF diets is the adaptive capacity of the SI. In the present study, we have observed differences in normal weight versus obese mice with respect to plasma TG appearance rates and satiety. Those results led us to speculate that the SI may be different based on chronic dietary consumption patterns. In order to test this theory, we placed female mice on either chow or high SFA diets for 12 weeks (*n* = 6 per group); the rationale was that chronic consumption of SFA may induce GI remodeling to reduce any potential deleterious effect of SFA. After 12 weeks, the animals were overweight (chow body weight = 29.7 ± 1.6 g vs. HF fed 38.3 ± 1.8 g; *p* < 0.0001). We tested the effect of oral gavage of a high SFA meal (Table 1) in chow and chronic high SFA-fed mice. We found that chronically feeding mice a high SFA diet results in elevated TG under fasting conditions as expected, followed by a relatively diminished postprandial peak (Figure 7A,B) with a lower peak and faster (sooner) reduction in plasma TG post-gavage. To better understand the mechanisms for these differences, we first measured total length of the small intestine (Figure 7C) which was significantly longer and cDNA expression of SCD1, Sterol Response Element-Binding Protein-1c (SREBP1c), and diacylglycerol acyltransferase-1 (DGAT1) (Figure 7D). SCD1 and SREBP1c were both increased along with the length of the SI suggesting that absorptive surface area, desaturation capacity, and lipogenic changes may induce a more rapid clearance of intestinal lipid contents.

**Figure 8:**
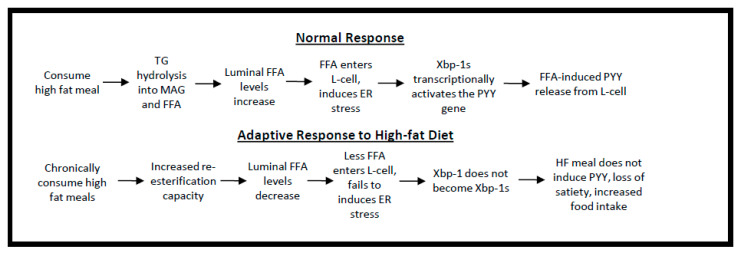
Conceptual Model. Dietary fat is ingested as triglyceride (TG) and digested into free fatty acids (FFA). The FFA’s in the intestinal lumen are potent inducers of satiety. However, the role of intestinal lipid synthesis is to re-esterify dietary FFA back into TG. The faster this happens, the less time FFA’s are available to induce satiety hormone production. A second aspect of the conceptual model is that FFA’s, especially saturated fatty acids (SFA), can cause a cell-stress like response by inducing X-box-binding protein 1 activation (Xbp1s). Xbp1s is a lipid sensitive transcription factor and the data suggest that it induces satiety hormone production. The faster rate of SFA desaturation to MUFA has two effects: (1) less SFA will reduce Xbp1s-mediated PYY production and (2) more MUFA will speed TG synthesis which will reduce FFA-mediated satiety leading to greater food intake and obesity.

Finally, we tested the postprandial PYY response of lean and obese mice given an SFA-rich oral gavage and the expected increase in PYY was observed in chow-fed mice after 40 min, which continued throughout the 2-hour test period. In HF-fed animals, the gavaged lipids resulted in a completely ablated postprandial PYY response (Figure 7E). Based on our preliminary data, we suspect that chronic SFA-rich diets lead to gut adaptations that increase desaturation and promote MUFA synthesis, which speeds TG production and clearance from the GI tract. The more rapid clearance prevents sufficient time to induce PYY gene expression in the L-cells and fails to induce satiety (Figure 8).

## 3. Discussion

We hypothesized that different FFA’s can directly affect the magnitude of PYY release based on differences in desaturation. This was based on classical work comparing absorption rates for dietary fat [22,23,24] with an initial study by Harry Steenbock and colleagues in 1936 [24] who found that absorption rates were much higher for linseed oil and olive oil (both high in MUFA and the slowest absorption rates were observed for coco butter, coconut oil, and palm oil (all high in SFA). Based on the composition of those fats, the oils with higher MUFA contents were being absorbed more quickly than the oils with higher SFA contents.

Several decades ago, Feldman et al. [22] measured lipoprotein and TG appearance in plasma in rats and showed that the rate of absorption of lipids was inversely associated with the percentage of SFA’s in the meal. More recently, Jackson et al. [25] compared HF meals that were rich in either palm oil (SFA-rich), safflower oil (PUFA-rich), a mixture of fish and safflower oil (PUFA-rich), and olive oil (MUFA-rich) on postprandial chylomicron levels in 10 postmenopausal women to better understand differences in rates of digestion and absorption between fats in humans. Chylomicron levels increased significantly more with olive oil (MUFA) vs. the other treatments. Their data indicates that MUFA-rich HF meals may result in faster dietary absorption than an SFA-rich HF meal. Based on the historical and current research, the present studies were based on the premise that SFA’s will take longer to absorb than MUFA’s and that the increased time the SFAs are in contact with cells in the GI tract will lead to extended PYY gene expression.

SFA’s are more slowly absorbed from the gut into the intestinal epithelial cells, possibly due to the fact that dietary SFA’s (or at least a portion of them) require desaturation prior to esterification [21]. The role of intestinal lipid synthesis is well-known and the contribution of TG anabolism during chylomicron synthesis has been established for many decades. Arguably, the change in lipogenic capacity during intestinal adaptation to HF diets may not be as well known. The few studies that have investigated the genetic, biochemical, and physiological changes in the SI with HF diets have consistently shown that the epithelial cell’s capacity for lipogenesis is significantly enhanced, most notably in the proximal and middle SI. One of the most notable changes is in SCD1 expression. SCD1 catalyzes the formation of unsaturated double bonds in long-chain SFA’s at the Δ9 position. As a result of chronic HF diets in mice, SCD1 upregulation in the duodenum is associated with increased TG appearance in plasma. Cross-sectional data from human models has shown that normal weight females increase plasma TG faster following a single high SFA meal compared to their lean counterparts and the obese females display a blunted satiety response. These data strongly imply that SFA’s satiety-inducing effects may be blunted with chronic consumption of SFA’s due to enhanced desaturase activity and increased lipogenic capacity of the intestine. If this is in fact the case, it may explain why obese individuals fail to receive sufficient satiety cues following meals.

The impact of lower satiety hormone production from MUFA may be due to increase food intake (feelings of hunger) which leads to increased body weight and obesity. Work from our lab and others has demonstrated that since MUFA’s are rapidly esterified into TG, they do not induce a cell stress-like response upon lipid loading [20,26]. Saturated fatty acids are more slowly esterified in the cell and they induce a robust cell stress response, seen as X-box-binding protein-1 splicing (Xbp1s). Among its various roles, the active Xbp1s protein is a lipid-induced transcription factor [16], and we found that it increases PYY gene expression in L-cells. Based on these previous studies, we hypothesized that that slower re-esterification of FFA’s into TG will increase satiety and decrease food intake. However, while we made every attempt to match the meal composition as closely as possible, there is a higher PUFA content in the MUFA meal which may confound the PYY response. Additional studies would be warranted to address each dietary fat source independently on PYY response in both normal weight and obese individuals.

The current studies demonstrate how satiety signaling is transmitted from dietary fat through the L-cell in normal and HF diet conditions. To date, very little is known regarding the mechanisms of nutrient–gene interactions in L-cells and these studies are some of the first of their kind. We assessed the factors involved in adaptive mechanisms of down-regulation of nutrient signaling in the gut and L-cells of HF-fed mice which can provide targets for therapeutic intervention as well as improved diet-design for individuals seeking to restrict calories to lose weight. We also determined the impact of ER stress on transducing nutrient signals into satiety hormone production in L-cells to better understand their adaptation to HF diets. The information provided from these studies helps to understand how dietary fatty acids are able to increase satiety hormone gene expression in the gut, and how obesity-associated loss of satiety occurs. Finally, it is important to consider dietary recommendations in which MUFA-rich diets are preferred over SFA-rich diets. However, this may result in weakened satiety signals and subsequently higher food intake.

## 4. Materials and Methods

### 4.1. Animals and Diets

All experiments using mice were approved by the Texas Tech University Institutional Animal Care and Use Committee (18 June 2012; 12052-06). Male and female C57BL/6 mice were purchased from Jackson Laboratories (Bar Harbor, ME, USA) and kept on a 12 h light/ dark cycle. Mouse research diets were from Harlan Laboratories (Madison, WI, USA); SFA (TD.130051) and MUFA (TD.130379) enriched diets were provided ad libitum for 4-weeks beginning at 8 weeks of age. For experiments involving diet-induced obesity, mice were either kept on standard rodent chow (TD.8604) or fed a high fat (HF) custom research diet (TD.06414) at 8 weeks of age for 12-weeks. Nutrient composition of the test diets are provided in Table 1.

### 4.2. Chemicals and Reagents

Sodium salts of FFA’s were purchased from Sigma (St. Louis, MO, USA). SCD1 (A939572) and Ire1α (4μ8c) inhibitors were from Calbiochem (EMD Millipore, Burlington, MA, USA). The processed murine SREBP1c coding sequence was amplified from liver cDNA, sub-cloned into pAdTrack-CMV, sequence verified, and incorporated into pAdEasy-1 vector via homologous recombination in BJ5183 cells (Agilent Technologies, Santa Clara, CA, USA). Total cellular lipids were extracted according to the Folch method [27] and resolved on a silica gel plate using hexane-diethyl ether-acetic acid (90:30:1) as the developing solvent. Mouse plasma PYY was measured by EIA (RayBiotech, Norcross, GA, USA) and human PYY was measured using RIA as previously described [19]. Plasma TGs were measured using the L-type triglyceride M kit (Wako Diagnostics).

### 4.3. In Vitro Studies

STC-1 cells were obtained from Dr. Douglas Hanahan via ATCC (Manassas, VA, USA) and grown in the recommended culture conditions (15% horse serum + 5% FBS, 5% CO_2_ and 1% antibiotic/1% antimycotic). The cells were grown to confluence then treated with unconjugated palmitate (16:0) or oleate (18:1) as FFA. In initial experiments, we found that BSA conjugated FFA’s do not induce PYY expression, yet the unconjugated Na-salts of FFA were effective. Cells were treated for indicated times in serum-free DMEM + 1% P/S to exclude effects from confounding lipids provided by horse or fetal bovine serum.

### 4.4. Oral Fat Tolerance Test

Six female mice were fasted for 4 h then received 0.2 mL of either the SFA- or MUFA-enriched oral gavage (Table 2). Five days later, the same animals received the other test meal. At baseline (0) and every 30-min for 4-h postprandial, blood samples were obtained via tail vein nick for measurement of TG via enzymatic assay (Wako Diagnostics).

### 4.5. Real-Time Quantitative PCR

Total RNA was isolated directly from cells or from frozen tissues using TRIreagent (Molecular Research Center, Cincinnati, OH, USA) and cDNA synthesis was performed using high capacity cDNA synthesis kits (Applied Biosystems, Foster City, CA, USA). Real-time PCR was performed using SYBR Green PCR Master Mix (Applied Biosystems). Gene-specific primers were designed using Integrated DNA Technologies software (www.IDTDNA.com) (Table 3) and all primers spanned exon-exon borders to eliminate the potential for genomic DNA amplification. Xbp1 splicing assay was performed as previously described [20].

### 4.6. Statistical Analyses

ANOVA or 2-tailed *t*-tests were performed using SAS software (SAS Institute, Cary, NC, USA). All values represent means ± SE of three independent experiments; * denotes a significant difference from the respective control (*p* < 0.05).

## Figures and Tables

**Table 1:** Composition of fat-enriched test meals.

	SFA	MUFA
Energy (kcals)	1.3	1.3
Energy from Fat (kcals)	1.0	0.9
Protein	15.8 mg	15.8 mg
Carbohydrate	75 mg	75 mg
SFA	58 mg	11 mg
MUFA	19 mg	60 mg
PUFA	7 mg	23 mg
% Energy from Fat	69.0	69.7
% Energy from test Fat	40.3	42.4

**Table 2:** Composition of fat-enriched diets.

Diet	Protein	CHO	Total Fat	SFA	MUFA	18:2n6	18:3n3	Kcal/g
Chow (TD.8604)	243	402	47	8	9	19	2	3.0
SFA (TD.130051)	177	330	222	530	330	27	10	4.0
MUFA (TD.130379)	177	330	222	120	670	14	5	4.0
High Fat (TD.06414)	235	273	343	370	470	160	10	5.1

**Table 3:** Primers used for PCR.

	Forward	Reverse
SCD1	AAGAGATCTCCAGTTCTTACA	GATATCCATAGAGATGCGCGG
SREBP1c	AGAAGCTCAAGCAGGAGAACCTGA	ACTTCGGGTTTCATGCCCTCCATA
PYY	TGCCTCTCCCTGTTTCTCGTATCC	AAGTCCACCTGTGTTCTCCTCCTC
CCK	TCAACTTAGCTGGACTGCAGCCTT	ACATACGCCGCTCTTCATGGCTTT
Xbp1	AAACAGAGTAGCAGCGCAGACTGC	TCCTTCTGGGTAGACCTCTGGGAG
DGAT1	CCTGAATTGGTGTGTGGTGATGCT	GCCAGGCGCTTCTCAATCTGAAAT
